# Preliminary exploration of acute limit toxicity testing for nicotinamide mononucleotide in the neonatal population

**DOI:** 10.3389/fphar.2026.1773471

**Published:** 2026-05-28

**Authors:** Hongting Cao, Xinyu Ge, Mingxuan Cui, Li Bao, Lijun Ma, Jinhai Gu, Ruijuan Xin, Guanghua Li, Li Bi, Fengchen Bi

**Affiliations:** 1 School of Public Health, Ningxia Medical University, Yinchuan, Ningxia, China; 2 School of Basic Medicine, Ningxia Medical University, Yinchuan, Ningxia, China; 3 The First Clinical Medical College, Ningxia Medical University, Yinchuan, Ningxia, China; 4 People’s Hospital of Ningxia Hui Autonomous Region, Ningxia Medical University, Yinchuan, China; 5 Key Laboratory of Cerebrocranial Disease, Ningxia Medical University, Yinchuan, Ningxia, China; 6 School of Information Engineering, Ningxia University, Yinchuan, China

**Keywords:** drug toxicity, neonates, nervous system, developmental toxicity, NMN

## Abstract

**Introduction:**

This study investigated the potential toxicity of nicotinamide mononucleotide (NMN) during the neonatal period, a critical and vulnerable developmental window. While NMN, a precursor of NAD^+^, has demonstrated safety and therapeutic potential in adults, its toxicological effects on newborns remain unclear.

**Methods:**

We conducted an acute limit toxicity test to evaluate the impact of NMN on vital organs (liver, kidney, and central nervous system) in neonatal mice.

**Results:**

Under these experimental conditions, no significant acute toxicity was observed, with no histopathological damage or serum biochemical abnormalities detected in the major organs. However, high-throughput transcriptome sequencing revealed subtle yet specific gene expression perturbations, involving liver proliferation signal regulation, kidney adaptation to excretory load, and brain suppression of neuroinflammation alongside coordinated growth and metabolism.

**Discussion:**

Despite the absence of overt histopathological or clinical toxicity, transcriptomic analysis revealed that a single high-dose NMN exposure may induce early molecular-level stress and compensatory adjustments in neonates. These findings provide crucial preliminary experimental evidence and transcriptomic insights for assessing NMN safety in early life, highlighting the need for future studies on long-term, subchronic, and chronic effects, while noting that the results cannot be directly extrapolated to human infants.

## 1 Introduction

The neonatal period is a critical life stage, during which drug administration and its toxicological evaluation are decisive for health outcomes. Compared to adults, neonates exhibit significant physiological differences, leading to distinct pharmacological characteristics and risks. Hepatic and renal functions are not fully mature; the activity of drug-metabolizing enzymes in the liver (particularly the CYP450 enzyme system) increases only gradually in the weeks after birth resulting in a reduced capacity for drug metabolism and excretion. Concurrently, body fluid composition, hemodynamics, and pharmacokinetic parameters differ significantly from those in adults (Ladumor et al., 2019). These factors collectively mean that neonates may experience more pronounced drug responses and a heightened risk of adverse effects. Furthermore, the neonatal immune system is not fully developed, which may increase susceptibility to immune-mediated adverse reactions, thereby elevating the potential for adverse outcomes (Taiorazova et al., 2023). Hence, developing individualized therapeutic approaches for this population is essential.

Nicotinamide mononucleotide (NMN) is a key precursor of nicotinamide adenine dinucleotide (NAD^+^). Its chemical structure confers excellent water solubility and favorable biocompatibility, enabling its efficient conversion to NAD^+^ *in vivo* (Ramanathan et al., 2022). NAD^+^ is a vital coenzyme and signaling molecule involved in fundamental processes such as energy production, DNA repair, gene expression, and cellular signal transduction, although its relatively large molecular size prevents direct cellular uptake. A growing body of research indicates that NMN holds therapeutic promise for various pathological conditions. It has shown potential in aging, neurodegenerative diseases such as Alzheimer’s disease (Ma et al., 2024) and Parkinson’s disease (Zhou et al., 2025), as well as in metabolic disorders and inflammatory injuries (Sabbagh and Kim, 2023), where it exerts protective effects. Clinical studies have demonstrated that oral NMN supplementation effectively increases plasma NAD^+^ levels and improves insulin sensitivity in healthy volunteers (Yamane et al., 2023). Additionally, NMN has been shown to modulate the gut microbiota, thereby influencing host physiology (Shi et al., 2025). Notably, NMN has demonstrated potential in neonatal diseases, such as providing neuroprotective effects in neonatal hypoxic-ischemic encephalopathy through modulation of the SIRT6-HMGB1 pathway (Kawamura et al., 2023).

In adult populations, NMN has demonstrated a favorable safety profile. Multiple randomized controlled trials have reported good tolerability of long-term NMN supplementation within established dosage ranges, and no serious adverse reactions have been documented (Song et al., 2023; Turner et al., 2021; Benjamin and Crews, 2024; Okabe et al., 2022). However, this existing safety evidence is almost exclusively derived from adult and elderly populations. Research on the pharmacokinetics, toxic effects, and potential side effects of NMN in newborns remains extremely limited, creating a significant knowledge gap that substantially hinders the clinical translation and application of NMN in neonatology. Therefore, this study aims to address this gap by conducting a preliminary evaluation of the potential toxicity of NMN in newborns, thereby providing essential preclinical evidence to inform its future safe application in this vulnerable population.

## 2 Materials and methods

### 2.1 Materials

#### 2.1.1 Experimental subjects

P9 C57BL/6J neonatal mice (body weight 4.5–6.5 g) were used in this study. The animals were housed under standardized conditions at a room temperature of 20 °C–22 °C, relative humidity of 50%–55%, and a 12 h light/dark cycle to minimize environmental stress. All neonatal mice were obtained from the Experimental Animal Center of Ningxia Medical University. All experimental protocols were approved by the Animal Ethics Committee of Ningxia Medical University (Protocol IACUC-2025376/2023-G251). At the endpoint, neonatal mice were euthanized by an overdose of isoflurane (5% for induction, maintained at 3% for 3 min) followed by cervical dislocation. Death was confirmed by the absence of respiration and cardiac arrest.

#### 2.1.2 Main reagents

The following reagents and antibodies were used in this study: β-Nicotinamide mononucleotide (NMN; purity ≥99%; CAS No.1094-61-7) was obtained from Shanghai E& Chemical Technology Co., Ltd. (China); Isoflurane was acquired from RWD Life Science Co., Ltd. (China). The anti-fluorescence quenching mountant containing DAPI and the tissue fixation solution were purchased from Beijing Labgic Technology Co., Ltd. (Biosharp, China). The Hematoxylin and Eosin (HE) staining kit was sourced from Wuhan Servicebio Technology Co., Ltd. (China). Primary antibodies included anti-MAP2 antibody (Abcam Inc., United States), NeuN Polyclonal antibody, and TAU Monoclonal antibody (both from Proteintech Group, Inc., China). Secondary antibodies were Alexa Fluor 555-Goat anti-chicken IgY (H + L) (biodragon, China), Alexa Fluor 488-labeled goat anti-rabbit IgG (Servicebio, China), and Cy3-labeled donkey anti-mouse IgG (Wuhan Savill Biotechnology Co., Ltd., China).

### 2.2 Methods

#### 2.2.1 Basis for route of administration and dose selection

The route of administration is a critical determinant of the metabolic fate and efficacy of NMN (Benjamin and Crews, 2024). Orally administered NMN undergoes extensive first-pass metabolism in the liver, where it is largely converted to nicotinamide (NAM), resulting in low systemic bioavailability and a tissue-restricted effect, predominantly elevating hepatic NAD^+^ levels. For neonatal mice, intravenous injection presents significant technical challenges, while intramuscular delivery is characterized by an extremely low absorption rate. To balance bioavailability with practical delivery efficiency, intraperitoneal injection was selected as the route of administration for this study. The dose was determined with reference to established guidelines for acute toxicity testing (OECD, 2022; EPA, 1998). A pilot study was first conducted on a single 9-day-old C57BL/6J neonatal mouse (an age corresponding to models of neonatal hypoxic-ischemic encephalopathy (Ma et al., 2024), administering 5,000 mg/kg NMN via intraperitoneal injection. No mortality was observed. Subsequently, two additional neonatal mice were tested under identical conditions, again with no mortality, leading to termination of the limit test. This preliminary finding indicated an LD_50_ greater than 5,000 mg/kg, suggesting low acute toxicity. Consequently, a dose of 5,000 mg/kg was employed for the main experimental investigation.

#### 2.2.2 Experimental animals and grouping

A total of twelve 9-day-old C57BL/6J neonatal mice were randomly divided into two groups. The treatment group received an intraperitoneal injection of NMN (5,000 mg/kg) dissolved in saline, which was freshly prepared prior to administration. The control group received an equal volume of saline vehicle. The total dose was administered as two separate intraperitoneal injections of 2,500 mg/kg each within a 24 h period. All animals were subsequently observed continuously for 14 days, during which their general health, behavior, and body weight were monitored and recorded.

#### 2.2.3 Serum biomarkers of hepatic function, renal function, and myocardial injury

On day 14 (the experimental endpoint), blood samples were collected from neonatal mice via the medial canthus and processed for biochemical analysis. Serum levels of liver function markers (alanine aminotransferase, ALT; aspartate aminotransferase, AST), renal function markers (creatinine, Cre; blood urea nitrogen, BUN), and cardiac injury markers (creatine kinase, CK; creatine kinase-MB, CK-MB) were measured.

#### 2.2.4 Hematoxylin and eosin (H&E) staining

Following the 14-day observation period after the dual intraperitoneal injections, the mice were sacrificed, and their visceral organs were harvested. The tissues were fixed overnight in 4% paraformaldehyde. Subsequent processing included dehydration through a graded ethanol series, clearing in xylene, and embedding in paraffin wax. Tissue sections (5 μm thick) were cut, mounted on slides, and dried at 65 °C for 1 h. For staining, the sections were first deparaffinized by immersion in xylene twice (10 min each) and then rehydrated through a descending ethanol series (100%, 90%, 80%, 70%; 5 min each). Hematoxylin staining was performed for 5 min with gentle vertical agitation to ensure uniformity, followed by a thorough rinse under running water. The sections were briefly differentiated in a hematoxylin differentiation solution (5 s), rinsed again, and then counterstained with eosin for 90 s. Finally, the sections were dehydrated through an ascending ethanol series, cleared in xylene, and mounted with neutral resin for microscopic observation.

#### 2.2.5 Immunofluorescence

Following anesthesia with isoflurane, neonatal mice were transcardially perfused, and their brains were harvested. The brains were fixed in 4% paraformaldehyde, cryoprotected in 30% sucrose, and embedded in optimal cutting temperature (OCT) compound. Frozen sections (5 μm thick) were cut and mounted on slides. For staining, sections were washed in PBS, subjected to antigen retrieval, and blocked with 5% normal goat serum for 1 h at room temperature. The sections were then incubated with primary antibodies overnight at 4°C, followed by incubation with appropriate fluorophore-conjugated secondary antibodies for 1 h at room temperature in the dark. Nuclei were counterstained with DAPI (1 μg/mL, 5 min). Finally, sections were mounted with anti-fade mounting medium and examined under a fluorescence microscope.

#### 2.2.6 Sample preparation and high - throughput sequencing

**FIGURE 1:**
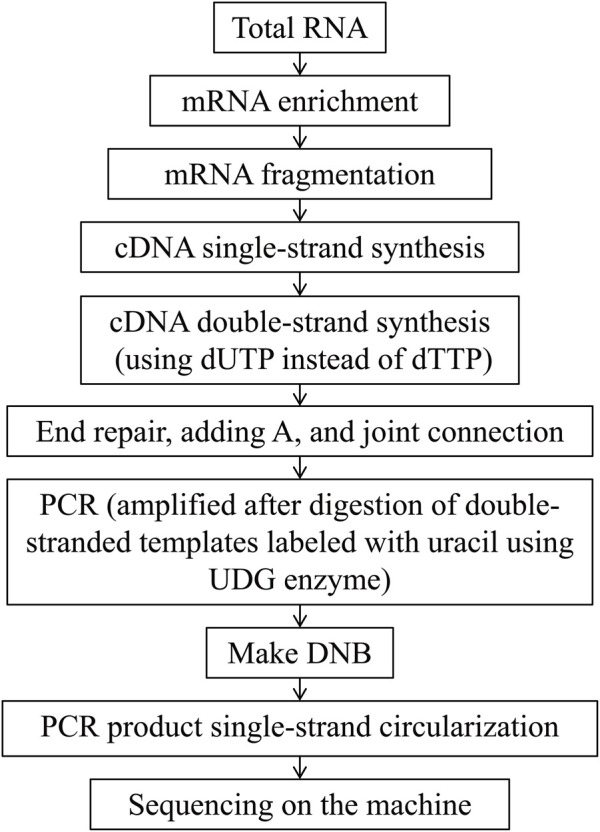
High-throughput sequencing workflow diagram.

Total RNA was extracted from tissue samples using a commercial kit (Thermo Fisher Scientific, Cat. No. 15596026CN). Transcriptome sequencing was performed by BGI using an Illumina platform. The reads were aligned to the mouse reference genome (GRCm38. p6, assembly accession GCF_000001635.26) using HISAT2 software. All sequenced libraries exhibited high-quality metrics, with Clean Reads Q20 scores exceeding 97% for every sample. A schematic diagram of the bioinformatics workflow is presented in the figure below, (Figure 1).

#### 2.2.7 High-throughput sequencing analysis

Raw sequencing data were first processed for quality control using fastp to obtain high-quality clean reads. These clean reads were then aligned to the reference genome with HISAT2, and transcript abundances were quantified using featureCounts. Differential gene expression analysis was performed with the DESeq2 package in R. Genes meeting the criteria of an absolute log2 fold change (|log2FC|) > 1 and an adjusted p-value (Benjamini–Hochberg) < 0.05 were identified as differentially expressed genes (DEGs). For visualization, volcano plots were generated using the ggplot2 package (R version 4.2.1) to summarize the differential expression results. Additionally, the outcomes of functional enrichment analyses (e.g., Gene Ontology or pathway analysis) were visualized using bubble plots.

#### 2.2.8 Statistical analysis

All statistical analyses were conducted using SPSS (version 27.0; IBM Corp.) and GraphPad Prism (version 10; GraphPad Software). Continuous data are presented as the mean ± standard deviation (mean ± SD). Differences between the two experimental groups were assessed using the unpaired Student’s t-test. A two-tailed *P* value of less than 0.05 was considered statistically significant.

## 3 Results

### 3.1 Acute toxicity limit assessment in neonatal mice

An acute toxicity limit test was performed using three 9-day-old C57BL/6J neonatal mice. All animals survived the 14-day observation period following the administration of a single 5,000 mg/kg dose of NMN, indicating a median lethal dose (LD_50_) greater than 5,000 mg/kg. No signs of acute toxicity were observed. These results are summarized in Table 1.

**TABLE 1:** Results of the acute toxicity limit test.

Animal id	Dose (mg/kg)	Survival status (14-day)
#1	5,000	Survived
#2	5,000	Survived
#3	5,000	Survived

All animals survived with no observable clinical signs of toxicity.

### 3.2 Clinical observations during acute toxicity assessment

During the 14-day observation period following NMN administration, all neonatal mice were closely monitored for signs of acute toxicity according to established observational criteria (Hayes and Kruger, 2014). As detailed in Table 2, no relevant clinical manifestations were observed across any of the specified physiological and behavioral indicators in the treatment group animals.

**TABLE 2:** Observation indicators for acute toxicity experiments.

Observation category	Specific indicators	Result summary (treatment group, n = 3)
I. Respiratory function	Dyspnea, abdominal breathing, wheezing, apnea, cyanosis, tachypnea, nasal discharge	All animals were negative (0/3)
II. Motor & neurological function	Changes in spontaneous activity, drowsiness, absence of reflexes, anesthesia, stagnation, motor incoordination, Abnormal movements Prone position Tremor, myocytic tremor	All animals were negative (0/3)
III. Convulsions (Seizures)	Clonic convulsions, tonic convulsions, Spastic-clonic seizures, syncope-induced convulsions, opisthotonos	All animals were negative (0/3)
IV. Reflexes	Corneal reflex, basic (pinna) reflex, positive (startle) reflex, tension reflex, pupillary light reflex	All animals were negative (0/3)
V. Ophthalmologic signs	Lacrimation, miosis, mydriasis, proptosis, ptosis, blood tears, relaxed eyelid, conjunctival/iris inflammation	All animals were negative (0/3)
VI. Cardiovascular signs	Bradycardia, tachycardia, vascular dilation, vasoconstriction, arrhythmia.	All animals were negative (0/3)
VII. Salivary secretion	Excessive salivation	All animals were negative (0/3)
VIII. Pilo-erection	Hair standing on end	All animals were negative (0/3)
IX. Analgesia	Decreased pain response	All animals were negative (0/3)
X. Muscle tone	Decreased or increased general muscle tone	All animals were negative (0/3)
XI. Gastrointestinal & renal signs	Constipation, diarrhea, vomiting, red urine, urinary incontinence	All animals were negative (0/3)
XII. Dermatological signs	Edema, erythema	All animals were negative (0/3)

All animals (n = 3) in the NMN-treated (5,000 mg/kg) group showed no adverse clinical signs across all monitored categories during the 14-day observation period.

### 3.3 Effect of NMN on body weight gain in neonatal mice

**FIGURE 2:**
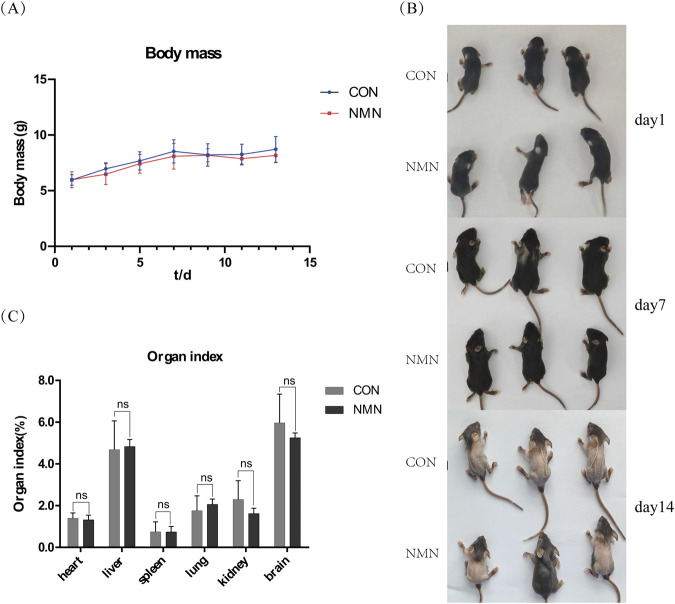
Effects of NMN on neonatal mice. **(A)** Body weight dynamics of control and NMN-treated neonatal mice over the 14-day observation period. No statistically significant differences in weight gain were observed between groups. **(B)** Representative images showing coat condition. Molting observed by day 14 was comparable between groups and consistent with natural postnatal molting. **(C)** Relative organ weights (organ coefficients) for heart, liver, spleen, lungs, and kidneys. No significant differences were found between the control and NMN-treated groups.

Body weight was measured at 48-h intervals from the first day of dosing (Day 1) until Day 13 (the end of the 14-day observation period). All groups of neonatal mice showed a steady increase in body weight over time, indicating normal postnatal growth. Statistical analysis confirmed that weight gain was comparable between the NMN-treated and the control group, with no statistically significant differences observed at any measured time point. These results are summarized in Table 3 and illustrated in Figure 2A.

**TABLE 3:** Weight changes in P9 neonatal mice during a 14-day drug administration period (g).

Animal id	DAY1	DAY3	DAY5	DAY7	DAY9	DAY11	DAY13
CON	6.30	7.70	6.80	7.70	7.90	8.10	8.80
	5.80	6.60	7.30	7.30	7.50	7.60	8.00
	6.20	7.30	8.80	9.50	9.60	9.50	10.40
	6.00	7.20	8.60	9.30	9.30	9.10	9.70
	6.40	6.80	7.20	9.60	7.10	7.00	7.30
	5.10	6.20	7.40	7.80	7.90	8.20	8.10
NMN	6.40	7.50	8.50	8.70	8.90	8.50	8.90
	5.40	6.40	7.80	7.90	8.10	7.80	8.00
	6.60	6.90	7.90	8.30	8.60	8.20	8.70
	5.90	4.80	6.10	6.70	8.50	7.10	7.20
	6.70	7.00	7.30	9.90	7.60	8.00	8.50
	4.90	6.30	6.90	7.10	7.40	7.70	7.70

### 3.4 Effects of NMN on physiological activity and coat condition in neonatal mice

A 14-day observation was conducted on 9-day-old C57BL/6J neonatal mice. The treatment group received a single high-dose intraperitoneal injection of NMN (5,000 mg/kg), while the control group received an equal volume of saline. Throughout this period, the mice were systematically monitored daily for key physiological and behavioral parameters, including: **alertness** and **reactivity** to environmental stimuli, **spontaneous activity** (e.g., foraging), **motor coordination** (gait, presence of tremors or ataxia), and **social/stress responses** (intra-cage interactions, response to handling). Body weight was regularly recorded as a measure of overall growth and metabolic status (reported in Section 3.3).

Throughout the observation period, no abnormalities were detected in any of the monitored physiological or behavioral parameters in either the control or NMN-treated groups, indicating sustained normal activity. Regarding coat condition, the fur of mice in both groups appeared smooth and glossy at the start of the experiment. By day 14, both groups exhibited molting (Figure 2B). Given the developmental stage of the animals, this phenomenon is consistent with a natural postnatal molting phase. Critically, the high-dose NMN intervention did not exacerbate or alter the pattern of molting compared to the control group.

### 3.5 Effect of NMN on relative organ weights in neonatal mice

Following the 14-day observation period, neonatal mice were humanely sacrificed and organs were harvested. The heart, liver, spleen, lungs, and kidneys were excised and weighed. The relative organ weight (organ coefficient) for each mouse was calculated as (organ weight/final body weight) × 100%. Statistical analysis revealed no significant differences in the relative weights of any of the examined organs between the NMN-treated and control groups (Table 4; Figure 2C).

**TABLE 4:** Changes in neonatal mice Organ Index (%).

Group	Animal id	Parameter Type	Heart	Liver	Spleen	Lung	Kidney	Brain
CON	1	Organ	0.09	0.336	0.05	0.171	0.173	0.417
		Body	7.4	7.4	7.4	7.4	7.4	7.4
		Index	1.22%	4.54%	0.68%	2.31%	2.34%	5.64%
	2	Organ	0.076	0.383	0.039	0.093	0.106	0.345
		Body	5.7	5.7	5.7	5.7	5.7	5.7
		Index	1.33%	6.72%	0.68%	1.63%	1.86%	6.05%
	3	Organ	0.071	0.129	0.021	0.115	0.162	0.364
		Body	4.4	4.4	4.4	4.4	4.4	4.4
		Index	1.61%	2.93%	0.48%	2.61%	3.68%	8.27%
	4	Organ	0.133	0.393	0.122	0.064	0.186	0.377
		Body	7.8	7.8	7.8	7.8	7.8	7.8
		Index	1.71%	5.04%	1.56%	0.82%	2.38%	4.83%
	5	Organ	0.079	0.301	0.023	0.103	0.089	0.363
		Body	7.2	7.2	7.2	7.2	7.2	7.2
		Index	1.10%	4.18%	0.32%	1.43%	1.24%	5.04%
NMN	1	Organ	0.081	0.358	0.03	0.137	0.107	0.351
		Body	6.7	6.7	6.7	6.7	6.7	6.7
		Index	1.21%	5.34%	0.45%	2.04%	1.60%	5.24%
	2	Organ	0.081	0.319	0.053	0.162	0.14	0.343
		Body	6.9	6.9	6.9	6.9	6.9	6.9
		Index	1.17%	4.62%	0.77%	2.35%	2.03%	4.97%
	3	Organ	0.09	0.389	0.061	0.183	0.137	0.425
		Body	8.3	8.3	8.3	8.3	8.3	8.3
		Index	1.08%	4.69%	0.73%	2.20%	1.65%	5.12%
	4	Organ	0.107	0.35	0.081	0.116	0.099	0.392
		Body	7	7	7	7	7	7
		Index	1.53%	5.00%	1.16%	1.66%	1.41%	5.60%
	5	Organ	0.097	0.274	0.035	0.125	0.084	0.324
		Body	6.1	6.1	6.1	6.1	6.1	6.1
		Index	1.59%	4.49%	0.57%	2.05%	1.38%	5.31%

### 3.6 Histopathological examination of visceral tissues

Histopathological analysis of the liver, kidney, heart, spleen, and lung tissues revealed no pathological alterations in either the control or NMN-treated groups.

**FIGURE 3:**
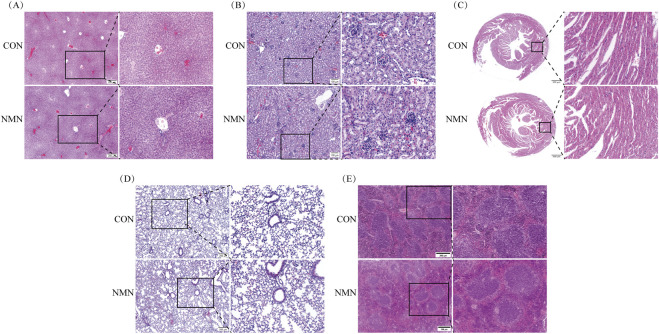
Histopathological analysis of major organs from neonatal mice. Representative hematoxylin and eosin **(H&E)**-stained sections of tissues from control and NMN-treated (5,000 mg/kg) groups are shown. No pathological alterations were observed in any of the examined organs following NMN treatment. **(A)** Liver tissue (20x magnification; scale bar = 200 µm). **(B)** Kidney tissue (20x magnification; scale bar = 100 µm). **(C)** Heart tissue (20x magnification; scale bar = 500 µm). **(D)** Spleen tissue (20x magnification; scale bar = 300 µm). **(E)** Lung tissue (20x magnification; scale bar = 200 µm).

Specifically, hepatic lobules showed a normal architecture with tightly packed hepatocytes and well-defined portal triads, without evident inflammatory infiltration, fibrosis, vacuolation, or pigment deposition (Figure 3A). In the kidney, glomeruli were intact without hyperplasia or inflammation, and renal tubules (both proximal and distal) displayed preserved epithelial structure with clear lumens (Figure 3B). Cardiac tissue exhibited regularly arranged cardiomyocytes with uniform nuclear staining and no evidence of fibrosis, atrophy, or necrosis (Figure 3C). Splenic architecture was maintained, with distinct white and red pulp areas showing no hyperplasia or erythrocyte stasis (Figure 3D). Pulmonary histology was unremarkable, featuring intact bronchioles and uniformly sized alveoli without dilation, collapse, inflammatory infiltration, congestion, or interstitial thickening (Figure 3E).

### 3.7 Serum biomarkers of hepatic function, renal function, and myocardial injury

On postnatal day 14, blood was collected from neonatal mice and subjected to biochemical analysis. The serum levels of key biomarkers, including liver function markers (ALT, AST), kidney function markers (creatinine, BUN), and myocardial injury markers (CK, CK-MB), showed no significant differences between the control and NMN-treated groups. All measured values remained within normal ranges, indicating no adverse effects on these organ systems following high-dose NMN administration. Detailed data are presented in Table 5.

**TABLE 5:** Serum biochemical parameters in P9 neonatal mice at day 14 following drug administration.

Parameter	Group	Value (mean ± SD)	References range	p-value
Liver function				
ALT (U/L)	CON	60.81 ± 12.95	10.06–96.47	>0.05
​	NMN	51.12 ± 6.14	​	​
AST (U/L)	CON	188.30 ± 27.24	36.31–235.48	>0.05
​	NMN	190.44 ± 7.55	​	​
Renal function				
Creatinine (μmol/L)	CON	23.14 ± 0.95	10.91–85.09	>0.05
​	NMN	23.83 ± 1.63	​	​
BUN (mg/dL)	CON	23.95 ± 0.33	10.81–34.74	>0.05
​	NMN	23.81 ± 0.50	​	​
Cardiac injury				
CK (U/L)	CON	881.78 ± 498.04	0–2070.55	>0.05
​	NMN	1244.62 ± 133.63	​	​
CK-MB (U/L)	CON	178.44 ± 62.17	1–1000	>0.05
​	NMN	223.63 ± 79.33	​	​

Data are presented as mean ± standard deviation (SD). Statistical analysis (e.g., Student’s t-test) showed no significant differences (p > 0.05) between the control and NMN-treated groups for any parameter. All values fell within the established physiological reference ranges. NMN, nicotinamide mononucleotide; ALT, alanine aminotransferase; AST, aspartate aminotransferase; BUN, blood urea nitrogen; CK, creatine kinase; CK-MB, creatine kinase-MB.

### 3.8 Histological assessment of brain tissue

**FIGURE 4:**
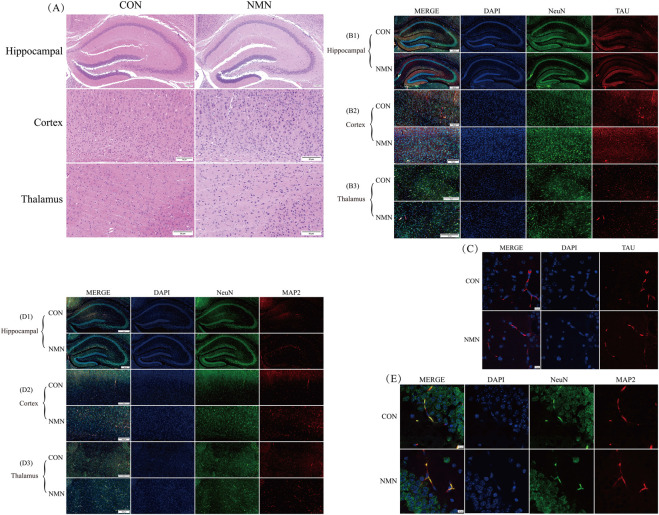
Histopathological and immunofluorescence analysis of brain tissue and synaptic markers in neonatal mice. Representative images from control and NMN-treated (5,000 mg/kg) groups demonstrate no observable morphological alterations following high-dose NMN intervention. **(A)** H&E-stained brain tissue section (20x magnification; scale bar = 50 µm). **(B1-C)** Immunofluorescence analysis of axonal integrity. Co-staining for the mature neuronal marker NeuN (green) and the axonal microtubule-associated protein Tau (red) in the **(B1)** hippocampus (scale bar = 100 µm), **(B2)** cortex (scale bar = 50 µm), and **(B3)** thalamus (scale bar = 100 µm). **(C)** High-magnification view (100x) of axonal morphology. **(D1-E)** Immunofluorescence analysis of dendritic structure. Co-staining for NeuN (green) and the dendritic marker MAP2 (red) in the **(D1)** hippocampus, **(D2)** cortex, and **(D3)** thalamus (scale bars = 100 µm). **(E)** High-magnification view (100x) of dendritic morphology.

Given the previously reported neuroprotective potential of NMN in developing nervous systems, we specifically evaluated its effects on central nervous system morphology. Hematoxylin and eosin (H&E) staining of brain tissue sections showed that neurons in both the control and NMN-treated groups were arranged in a well-organized and orderly manner, with uniform cellular morphology and homogeneous chromatin staining. No pathological alterations were observed (Figure 4A).

### 3.9 Evaluation of neuronal synaptic morphology

The impact of high-dose NMN on synaptic structures was assessed through detailed morphological analysis of axons and dendrites.

#### 3.9.1 Effect of NMN on neuronal axons

The expression and distribution of the mature neuronal marker NeuN and the axonal microtubule-associated protein Tau were analyzed by immunofluorescence. No significant differences were found between the NMN-treated and control groups in the number, soma morphology, or distribution density of NeuN-positive neurons in the cerebral cortex. Tau protein exhibited a typical intracellular distribution pattern, localized to neuronal cell bodies and specifically enriched in axonal domains. The fluorescence signal intensity and spatial distribution of Tau were comparable between the two groups (Figures 4B,C). These results indicate that, under the present experimental conditions, NMN did not affect neuronal survival or axonal structural integrity.

#### 3.9.2 Effects of NMN on neuronal dendrites

Immunofluorescence co-localization analysis of neurons (NeuN) and the dendritic marker microtubule-associated protein 2 (MAP2) was performed. In both groups, MAP2 signals were clearly distributed around the soma of NeuN-positive neurons, displaying typical bundled fiber structures that outlined complete dendritic arbors (Figures 4D,E). Comparison between groups revealed no significant differences in MAP2 expression intensity, distribution range, or fiber morphology. This suggests that high-dose NMN intervention did not induce observable alterations in neuronal dendritic structure.

Integrated analysis of both axonal (Tau) and dendritic (MAP2) markers confirmed the absence of significant morphological damage to synaptic structures in cortical neurons following acute high-dose NMN exposure. Collectively, from a histomorphological perspective, these data support the preliminary conclusion of a high safety profile for NMN in the neonatal nervous system.

### 3.10 Transcriptomic profiling of liver, kidney, and brain tissues

To assess potential molecular-level effects, RNA sequencing (RNA-seq) was performed on liver, kidney, and brain tissues from NMN-treated and control neonatal mice.

#### 3.10.1 Sequencing data quality and overview

A total of 18 samples (n = 3 per organ per group) were sequenced. The data output demonstrated high quality: each sample yielded approximately 6.63 Gb of data on average; the mean alignment efficiency to the reference genome was 99.15%, and to the known gene set was 73.93%. These metrics confirm high data quality and reliability for downstream analyses. A total of 18,895 genes were identified as expressed across all samples.

#### 3.10.2 Identification of differentially expressed genes

**FIGURE 5:**
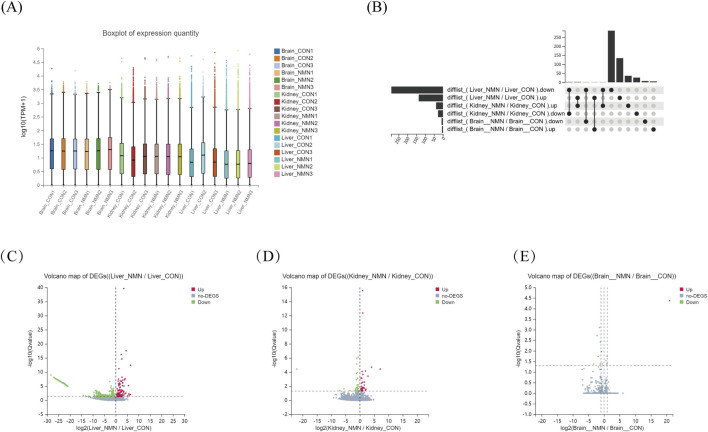
Transcriptomic overview and identification of differentially expressed genes (DEGs) in liver, kidney, and brain tissues. Comparative RNA-seq analysis of control and NMN-treated neonatal mice reveals organ-specific transcriptional responses to NMN. **(A)** Distribution of gene expression levels across all samples. **(B)** Venn diagram depicting the number of unique and shared DEGs (*P* < 0.05) among the three organs. **(C–E)** Volcano plots visualizing DEGs in the **(C)** liver, **(D)** kidney, and **(E)** brain for the control (CON) *versus* NMN-treated comparison (*P* < 0.05).

Initial differential expression analysis (|log2FC| > 0 & P < 0.05) indicated that NMN administration elicited transcriptomic alterations in multiple organs, with varying numbers of differentially expressed genes (DEGs) identified in the liver, kidney, and brain, respectively (Figure 5). A set of core DEGs was selected for further analysis. Specifically:

**Liver**: Using |Log_2_FC| > 2 and P < 0.05, **12 core DEGs** were selected for further study.

**Kidney**: Based on |Log_2_FC| > 2 and P < 0.05, **11 core DEGs** were chosen to further analysis.

**FIGURE 6:**
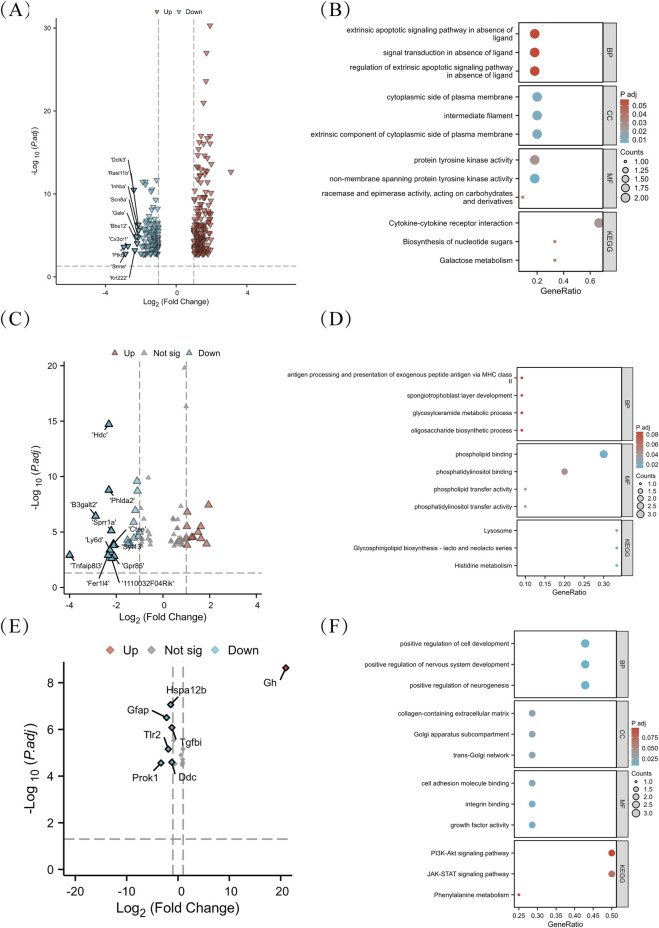
Identification of core differentially expressed genes (DEGs) and their functional enrichment in liver, kidney, and brain tissues. **(A,C,E)** Volcano plots identify core DEGs in the **(A)** liver (|Log_2_FC| > 2, *P < 0.05*), **(C)** kidney (|Log_2_FC| > 2, *P < 0.05*), and **(E)** brain (|Log_2_FC| > 1, *P < 0.05*) for the control *versus* NMN-treated comparison. **(B,D,F)** Bubble plots of Gene Ontology (GO)/KEGG enrichment analysis for the core DEGs in **(B)** liver, **(D)** kidney, and **(F)** brain tissues, highlighting significantly enriched biological pathways.

**Brain**: Applying |Log_2_FC| > 1 and P < 0.05, **7 core DEGs** were identified (considering that gene expression changes in neural tissue are typically of lower magnitude, and given the very limited total number of DEGs identified in the brain from the initial analysis, a more relaxed fold-change threshold (|Log2FC| > 1 and P < 0.05) was applied.) (Figure 6).

#### 3.10.3 Functional enrichment analysis of core DEGs

Gene Ontology (GO) and KEGG pathway enrichment analyses were performed on the core DEGs to elucidate their potential biological functions. The results revealed that these genes were not randomly distributed but showed significant enrichment in specific biological processes (Figure 6):

Liver: DEGs were predominantly enriched in terms related to **non-membrane-spanning protein tyrosine kinase activity** (e.g., Ptk6), **the cytoplasmic side of the plasma membrane** (e.g., Ptk6), and **intermediate filament organization** (e.g., Fbf1, Krt222).

Kidney: DEGs (e.g., PHLDA2, Tnfaip8l3, Syt13) were significantly enriched for terms related to **phospholipid binding**, suggesting potential adaptive changes in cell membrane-related signaling and transport.

Brain: DEGs (e.g., GFAP, TLR2, GH) were mainly enriched in pathways associated with **nervous system development** and cellular regulation. Collectively, these enrichment results indicate organ-specific transcriptomic responses to high-dose NMN, without evidence of overt pathological or toxicological pathway activation, consistent with the observed safety profile.

## 4 Discussion

The promising therapeutic potential of NMN necessitates a thorough safety evaluation, a critical prerequisite for its clinical translation. Accumulating clinical evidence indicates favorable safety and tolerability of NMN in adults. Research by Klein and Yoshino (2021) provided initial evidence that a 10-week oral regimen of 250 mg/day NMN significantly improved insulin sensitivity in postmenopausal women with prediabetes. Importantly, the study reported no significant toxic side effects or clinically relevant changes in serum biochemistry, blood lipids, or blood pressure compared to the placebo group, supporting its safety in this cohort. A subsequent randomized, double-blind, placebo-controlled trial (Fukamizu et al., 2022) conducted a more systematic assessment. Healthy adults received up to 1,250 mg of NMN daily for 4 weeks. The results confirmed that even at this higher dose, NMN was well-tolerated with no serious drug-related adverse events. Comprehensive clinical examinations, including blood tests and urinalysis, showed all parameters remained within normal physiological ranges. In summary, clinical studies spanning from individuals with prediabetes to healthy adults, and employing doses from conventional to high levels, consistently report excellent tolerability and a reliable safety profile for NMN in adult participants over short-to-mid-term administration.

The therapeutic potential of nicotinamide mononucleotide (NMN) extends to neonatal medicine, with emerging evidence suggesting its utility in conditions such as hypoxic-ischemic encephalopathy (HIE). Studies in neonatal HIE model mice demonstrate that NMN administration rapidly restores hippocampal NAD^+^ and SIRT6 levels, effectively inhibiting apoptotic and inflammatory pathways (caspase-3, HMGB1), leading to significant reductions in brain tissue loss and improved neurobehavioral outcomes (Kawamura et al., 2023). Given that the current standard therapy for HIE, therapeutic hypothermia, is limited by a narrow treatment window and high resource demands (Zou et al., 2019), NMN presents a promising candidate strategy to potentially extend this critical window.

However, a systematic evaluation of NMN safety in the vulnerable neonatal population remains a critical unmet need. Neonates possess distinct and immature physiological and metabolic profiles, particularly in liver and kidney function, which markedly reduce drug metabolism and clearance capacities (Roberts et al., 2021). This immaturity predisposes them to drug accumulation and altered toxicological responses, as evidenced by differing adverse event spectra for various common medications compared to adults (Byskov et al., 2024). Consequently, safety assessments for neonatal therapeutics must be specifically tailored and cannot be extrapolated from adult data.

To address this gap, our study represents the first investigation employing an acute limit test, a design specifically suited for initial safety screening in newborns, to evaluate the response to high-dose NMN exposure. This work provides a crucial preliminary safety profile, establishing a foundational benchmark for NMN in neonates. This initial step is essential for designing subsequent, more comprehensive toxicological studies involving multiple doses and extended durations, ultimately aiming to build a robust safety evaluation framework to support potential clinical translation.

### 4.1 Study design and dose rationale

This study was conducted within the acute toxicity testing framework, involving single or multiple administrations within 24 h, followed by a 7–14 day observation period to assess acute toxic effects and inform clinical safety (Wang et al., 2022). To model the neonatal stage, 9-day-old C57BL/6J neonatal mice were used.

Dose selection followed the “limit test” procedure specified in international guidelines (EPA, 1998; OECD, 2022), which is recommended for substances with anticipated low toxicity based on existing data. As NMN has shown no significant toxicity in adult studies, this criterion was met. In a preliminary experiment, three neonatal mice received an intraperitoneal dose of 5,000 mg/kg NMN; all survived without signs of intoxication, indicating a median lethal dose (LD_50_) >5,000 mg/kg. This dose was therefore selected as the maximum tolerated dose for subsequent toxicity characterization.

Deviating from the traditional multi-dose design, this study employed a limit-test approach, using only a control group and the 5,000 mg/kg NMN group. This strategy is justified because (1) prior clinical data in adults support the low toxicity of NMN, and (2) for low-toxicity compounds, a limit test can effectively identify potential target organs of toxicity while significantly reducing animal use, aligning with the “reduction” principle of animal ethics.

### 4.2 Rationale for split-dose administration within 24 hours

The design of administering the total dose in two fractions (administered in two equal fractions of 2,500 mg/kg) within 24 h was informed by the distinct pharmacokinetic profile of neonates. The hepatic cytochrome P450 (CYP450) system is immature (Goldman and Cheng, 2025), and renal glomerular filtration rate (GFR) only gradually reaches adult levels during the first postnatal year (Iacobelli and Guignard, 2021). Consequently, the combined limited hepatic and renal clearance capacity in newborns raises a critical concern: a single bolus of a very high dose (5,000 mg/kg) could produce excessively high peak plasma concentrations, potentially leading to non-specific, concentration-dependent toxicity that could mask the true biological or toxicological effects of NMN. To mitigate this risk and better model a sustained high-level exposure scenario—which is more relevant to potential clinical use—the total dose was divided. This split-dose regimen aimed to achieve a more prolonged and pharmacologically realistic exposure profile, thereby enabling a more accurate and gentle assessment of the organism’s response to high-dose NMN under conditions that approximate a continuous therapeutic challenge.

### 4.3 Rationale for intraperitoneal administration

While the systematic toxicology of NMN in neonatal mice remains uncharacterized, its safety profile is strongly supported by extensive data from adult animal models. Studies confirm that intraperitoneal injection (i.p.) of NMN (e.g., 500 mg/kg) effectively mitigates age-related functional decline, enhances neurovascular health and insulin secretion, and improves vascular compliance, with no acute toxic effects reported (Nadeeshani et al., 2021). Notably, even a single high-dose intravenous (i.v.) bolus of 2000 mg/kg resulted in no mortality or abnormal clinical signs during a 14-day observation period (Song et al., 2023). Collectively, these findings underscore that NMN, as an endogenous compound, possesses very low acute toxicity and a wide safety margin, supporting its use in acute exposure studies.

In this study, a high intraperitoneal dose of 5,000 mg/kg was employed to investigate the acute toxicity limit of nicotinamide mononucleotide (NMN). Consistent with prior literature and our preliminary data, NMN demonstrates a wide safety margin in acute exposure. The i. p. route was selected specifically to establish a proof-of-concept, high-systemic-exposure model for assessing intrinsic organ toxicity. This design intentionally circumvents the confounding variables of first-pass metabolism, thereby enabling more direct extrapolation of findings for human safety assessment.

We acknowledge that intraperitoneal administration is not a standard clinical route. Its selection was driven by a combination of practical necessity and scientific rationale specific to neonatal rodent models. Technically, oral gavage and intravenous injection in neonatal mice are highly challenging and associated with significant procedural mortality. Therefore, the i.p. route was chosen to minimize animal loss due to technical artifacts. From a methodological standpoint, i. p. injection is a well-established paradigm in fundamental toxicology for the preliminary evaluation of systemic acute toxicity. It ensures accurate dosing and facilitates rapid, nearly complete systemic absorption. This allows the study to focus on the direct effects of NMN at extremely high circulatory concentrations, effectively controlling for the complex and immature variables of neonatal intestinal absorption and hepatic metabolism, and providing a “worst-case” exposure scenario to understand inherent toxicity potential. Furthermore, utilizing the i. p. route enhances the comparability and logical coherence of our argument, as most key prior studies establishing high-dose NMN safety, including those in neonatal mice, have employed injection-based (i.p. or i. v.) methodologies.

The value of this i. p. Model data lies in its role as a pivotal node in risk assessment, not as a direct clinical surrogate. The observation of no acute organ injury under this high-exposure, first-pass-avoidant model provides robust support for NMN’s broad therapeutic index, indicating a low risk of direct organ toxicity even at supraphysiological systemic levels. While pharmacokinetic profiles differ, the “no-observed-adverse-effect” dose (NOAEL) identified here provides a vital parameter. Furthermore, the early stress response pathways revealed by transcriptomics furnish additional data for constructing subsequent physiologically based toxicokinetic (PBTK) models. These models are indispensable for predicting safety boundaries under various clinical dosing regimens.

In conclusion, while this study delivers crucial acute safety data, its full translation necessitates more comprehensive evaluation. Future research must expand to encompass alternative administration routes, extended observation periods, a broader panel of biomarkers, and chronic toxicity assessments. These efforts should systematically integrate evaluations of growth, neurodevelopment, reproductive function, and long-term metabolic outcomes to ultimately define a safe therapeutic protocol for human newborns.

### 4.4 Histological and biochemical integrity following high-dose exposure

The status of neonatal mice was monitored continuously for 14 days following the acute exposure. Body weights were recorded on days 1, 3, 5, 7, 9, 11, and 13. The results showed that both groups exhibited normal growth trajectories, with no significant intergroup differences. Throughout the observation period, basic physiological activities—including fur condition, spontaneous movement, and feeding behavior—remained normal in both control and NMN-treated pups. On day 14, blood was collected terminally, and mice were euthanized for visceral examination. Histopathological (H&E staining) and serum biochemical findings were consistent, revealing no observable injury to major organs following acute high-dose (5,000 mg/kg) NMN exposure. Specifically, hepatocytes were tightly arranged with regular morphology, and serum alanine aminotransferase (ALT) and aspartate aminotransferase (AST) levels fell within the normal range. Glomerular architecture was clear, renal tubules were intact, and serum creatinine (Cr) and blood urea nitrogen (BUN) levels were normal. Myocardial cells displayed a clear, orderly structure, accompanied by normal serum creatine kinase (CK) and its MB isoenzyme (CK-MB) levels. No abnormalities were noted in the spleen or lung. Collectively, these data confirm that NMN did not induce acute structural or functional damage to the liver, kidney, heart, or other vital organs in neonatal mice under the experimental conditions.

### 4.5 Rationale for selecting the 14-day terminal timepoint

The 14-day timepoint for terminal analysis was selected to avoid potential false-positive signals arising from neonatal metabolic immaturity. Although adult studies indicate that acute injury markers (e.g., transaminase peaks) typically appear within 24–72 h post-exposure for drugs with pronounced hepatotoxicity such as paracetamol (Chidiac et al., 2023), newborns present a distinct physiological state. Hepatic drug-metabolizing enzymes (e.g., CYP450) in neonatal mice—and human neonates—are markedly underdeveloped, with activity substantially lower than in adults, leading to significantly delayed drug clearance (Goldman and Cheng, 2025). If testing were performed during the early exposure phase (e.g., 24–72 h), non-specific, transient biochemical fluctuations driven by the accumulation of the compound and its metabolites could be misinterpreted as toxicological signals, yielding “false-positive” outcomes. Moreover, this design aligns with histopathological and transcriptomic endpoints, allowing the capture of sustained or adaptive changes rather than transient peaks. The core objective was not to capture fleeting acute-injury peaks, but to evaluate the post-acute adaptive state of the organism after NMN exposure—particularly following initial metabolic adaptation—and to establish a spatiotemporal correlation with concurrent histopathology and multi-organ transcriptomics, thereby enabling a systematic dissection of NMN’s biological effects.

As a limitation of this preliminary study using terminal blood collection, early dynamic pharmacokinetic data were not obtained. Consequently, future investigations will incorporate serial non-terminal blood-sampling protocols, enabling dynamic monitoring of biochemical and hematological indices at multiple timepoints (e.g., 24, 48, 72 h and beyond). This approach should help to: (1) clarify the early pharmacokinetic and pharmacodynamic profile of NMN in neonates; (2) distinguish between transient metabolic adaptations and genuine progressive toxic injury; (3) construct a more comprehensive, time-resolved safety-assessment map, providing precise data for defining the compound’s therapeutic window.

### 4.6 Liver transcriptomic profiling and mechanistic insights

Following stringent screening (|log2FC| > 2 and P < 0.05), 12 differentially expressed genes (DEGs) were chosen for subsequent analysis. Gene Ontology (GO) and Kyoto Encyclopedia of Genes and Genomes (KEGG) enrichment analyses of these DEGs indicated they were primarily associated with functions and localizations related to non-membrane spanning protein tyrosine kinase activity, the extrinsic component of the cytoplasmic side of the plasma membrane, and intermediate filament organization.

The enrichment was largely driven by genes encoding Src-related kinase lacking C-terminal regulatory tyrosine and N-terminal myristoylation sites (SRMS), protein tyrosine kinase 6 (PTK6), keratin 222 (KRT222), and fibrous sheath-interacting protein 1 (FBF1). This pattern suggests that acute high-dose NMN exposure perturbs signaling pathways centered on non-receptor tyrosine kinases (e.g., SRMS, PTK6) and influence cytoskeletal structure (via KRT222, FBF1) in the neonatal liver, pointing to specific adaptive or stress-responsive molecular pathways rather than overt toxic injury.

### 4.7 Mechanistic implications of the coordinated downregulation of SRMS and PTK6

The coordinated downregulation of two Src-family kinases, SRMS and PTK6, in the neonatal liver following high-dose NMN exposure points to a specific modulation of tyrosine kinase signaling pathways. SRMS (Src-related kinase lacking C-terminal regulatory tyrosine and N-terminal myristoylation sites) is a non-receptor tyrosine kinase whose function appears non-essential under basal conditions, as SRMS-deficient mice display no overt phenotypes, suggesting functional redundancy within the kinase family (Goel et al., 2023; McClendon and Miller, 2020). PTK6 (Protein tyrosine kinase 6) is recognized for its context-dependent oncogenic roles; in the liver, its elevated expression promotes hepatocyte proliferation and tumorigenesis, whereas its suppression can exert a protective, anti-proliferative effect (Chen et al., 2016). The observed downregulation of PTK6 here hints at a potential chemopreventive role for NMN in mitigating excessive proliferative signaling.

The synergistic decrease in both kinases suggests that NAD^+^ overload may broadly attenuate specific branches of tyrosine kinase signaling. This hypothesis is supported by prior evidence that NMN administration can alter the phosphorylation status of the Src/PI3K/Akt pathway—a major downstream axis of non-receptor tyrosine kinases—in other cell types under stress (Liu et al., 2025). Therefore, we speculate that the NMN-induced NAD^+^ surplus in neonatal mice may lead to reduced phosphorylation/activity of Src-family kinases, resulting in the transcriptional downregulation of PTK6 and SRMS and consequent inhibition of downstream PI3K/Akt-mediated regulatory networks. This mechanism aligns with NMN’s known role in reducing oxidative stress and DNA damage, positioning it as a modulator of stress-responsive kinase cascades.

### 4.8 Biological implications of altered FBF1 and KRT222 expression

The observed upregulation of FBF1 (fibrous sheath interacting protein 1) and downregulation of KRT222 (keratin 222) in the neonatal liver following NMN exposure point to a coordinated cellular adaptation involving cell survival and cytoskeletal remodeling.

FBF1 is a cilia-associated protein implicated in signal transduction, with highly restricted expression in normal tissues, primarily within germ cells (Zhang et al., 2025). Its downregulation has been linked to promoting apoptosis in damaged hepatocytes, facilitating tissue turnover and functional recovery. Given that FBF1 downregulation promotes apoptosis, its observed upregulation here suggests a potential anti-apoptotic, hepatoprotective effect of NMN. KRT222 is a member of the keratin family, key components of the epithelial cytoskeleton that regulate cell mechanics, migration, and differentiation (Köster and Herrmann, 2026). The downregulation of KRT222 likely indicates a decrease in hepatocyte stiffness, representing an active remodeling of the cytoskeleton in response to metabolic stress.

This pattern appears to contrast with prior studies showing NMN protects against cytoskeletal damage (Nakajo et al., 2024). The discrepancy may be explained by several critical factors specific to our experimental design: (1) Dose disparity: Most adult studies use lower, protective doses (Nadeeshani et al., 2021), whereas our extreme dose (5,000 mg/kg) may trigger a distinct stress-regulatory response. (2) Physiological state: The neonatal liver is in a highly proliferative and differentiation-active phase, potentially responding to NAD^+^ flux differently than a quiescent adult liver. (3) Exposure paradigm: Previous findings often result from chronic adaptation, whereas our acute exposure model captures the early stress response prior to potential long-term adaptation.

Mechanistically, these changes may be orchestrated through the SIRT1-AMPK signaling axis, a key pathway influenced by the NAD^+^/NADH ratio. AMPK, a direct energy sensor and target of SIRT1 (Zhao et al., 2012), phosphorylates numerous cytoskeletal proteins (Crosas-Molist et al., 2023). The altered NAD^+^ pool from NMN may thus compensatorily regulate KRT222 and FBF1 expression via SIRT1-AMPK activation, driving the observed cytoskeletal and survival adjustments. These insights carry direct translational implications: NMN should be used cautiously in neonates with pre-existing hepatocyte injury, and clinical monitoring should consider parameters related to liver mechanics and function, such as hepatic vascular pressure.

### 4.9 Renal transcriptomic profiling and molecular insights

To evaluate the potential molecular impact of NMN on the neonatal kidney, high-throughput RNA sequencing was performed on renal tissues from control and NMN-treated neonatal mice. Following the initial screening, a set of 11 DEGs meeting the threshold (|log2FC| > 2 and P < 0.05) was chosen for subsequent analysis. GO and KEGG enrichment analyses of these DEGs showed significant enrichment for the biological process of phospholipid binding.

This enrichment was primarily driven by three key genes: pleckstrin homology-like domain family A member 2 (PHLDA2), tumor necrosis factor alpha-induced protein 8-like 3 (TNFaip8l3), and synaptotagmin 13 (SYT13). The coordinated alteration of these genes suggests that acute high-dose NMN exposure may perturb cellular signaling or metabolic pathways related to phospholipid membrane dynamics in the developing kidney, pointing to a specific adaptive response in renal tissue.

### 4.10 Mechanistic implications of PHLDA2 downregulation

PHLDA2 (pleckstrin homology-like domain family A member 2) is an imprinted gene that functions as a critical regulator of apoptosis. Evidence indicates that PHLDA2 promotes cellular senescence and apoptosis by inducing mitochondrial dysfunction via activation of the Wnt/β-catenin pathway, whereas its knockdown improves mitochondrial membrane potential and inhibits these processes (Chang et al., 2024). It also serves as an independent high-risk prognostic marker in metastatic clear cell renal cell carcinoma. The downregulation of PHLDA2 observed here following NMN intervention suggests a potential renoprotective effect, possibly through the enhancement of mitochondrial function and suppression of apoptotic pathways.

This regulatory effect may operate through multiple interconnected mechanisms. First, high-dose NAD^+^ precursors can influence cellular methyl metabolism, altering DNA and protein methylation states (Hwang and Song, 2020). The NMN-induced NAD^+^ overload could thus deplete methyl donors, potentially affecting the epigenetic regulation of the imprinted PHLDA2 locus. Second, PHLDA2 is known to act as a negative regulator of the mTOR pathway; its downregulation can inhibit the AKT/mTOR axis, thereby modulating cell proliferation and death (Guo et al., 2022), Given that elevated NAD^+^ levels are known to suppress mTOR signaling via SIRT1 activation (Lu et al., 2021), we speculate that NMN-mediated NAD^+^ surplus may reduce the cellular demand for the negative feedback regulator PHLDA2 through SIRT1-dependent mTOR inhibition, leading to its transcriptional downregulation.

In summary, the altered expression of PHLDA2 not only provides a molecular clue for NMN’s potential to improve renal mitochondrial health but also highlights the involvement of the NAD^+^/SIRT1/mTOR signaling axis in mediating this adaptive response.

### 4.11 Mechanistic implications of Tnfaip8l3 downregulation

Tnfaip8l3 (TNFα inducible protein 8-like 3) is a member of the TNFAIP8/TIPE family, crucial for regulating inflammatory responses and immune homeostasis (Li et al., 2021). Its dysregulated expression is implicated in disease progression; notably, Tnfaip8l3 is upregulated in mesangial cells and animal models of diabetic nephropathy (Bordoloi et al., 2018). The downregulation of Tnfaip8l3 observed in the neonatal kidney following NMN intervention suggests a potential protective role of NMN in mitigating inflammatory pathways associated with renal pathology, such as diabetic nephropathy. The underlying mechanism likely involves the SIRT1/NF-κB signaling axis. SIRT1 deacetylates the RelA/p65 subunit of NF-κB, thereby inhibiting its transcriptional activity (Wang et al., 2024). The NMN-induced NAD^+^ surplus may potentiate this SIRT1-mediated inhibition, leading to reduced basal expression of downstream NF-κB target genes, including Tnfaip8l3. Thus, the alteration in Tnfaip8l3 expression not only reflects the impact of NAD^+^ flux via the SIRT1/NF-κB pathway but may also signify an adaptive recalibration of renal inflammatory and energy metabolic states in response to acute high-dose exposure.

### 4.12 Mechanistic implications of Syt13 downregulation

Syt13 (synaptotagmin 13) is a member of the synaptotagmin family, primarily involved in calcium-dependent vesicle trafficking and cellular signal transduction. Current literature predominantly describes its roles in the nervous system (Miki et al., 2025), cancer (Zhang et al., 2023), and endocrine system (Bakhti et al., 2022; Ofori et al., 2022), with no direct studies linking it to renal function. However, based on renal tissue architecture and Syt13’s molecular function, we propose a plausible role in the kidney. The kidney is a highly polarized epithelial tissue whose reabsorptive and secretory functions critically depend on precise membrane transport systems. Specialized structures, such as the macula densa in the juxtaglomerular apparatus—a “synapse-like” chemoreceptor—sense changes in tubular fluid composition. We speculate that Syt13 may participate in vesicular release or intercellular signaling within such structures, thereby influencing tubular functions like NaCl reabsorption. Consequently, NMN intervention warrants attention to indices of tubular function.

Mechanistically, this regulation may involve calcium signaling pathways. cADPR (cyclic adenosine diphosphate ribose), a metabolite of NAD^+^, is a key intracellular calcium-mobilizing messenger (Kim, 2022). High-dose NMN could elevate cADPR levels, perturbing intracellular calcium homeostasis (Zhu et al., 2025) and triggering feedback regulation of calcium-sensing proteins like Syt13. Thus, the observed Syt13 downregulation likely reflects an adaptive transcriptional adjustment by renal tubular epithelia to high metabolic and excretory demands, fine-tuning vesicle transport and calcium signaling systems.

### 4.13 Integrated interpretation and phenotypic dissociation

Collectively, the transcriptomic changes suggest that NMN may confer potential renoprotective effects—enhancing mitochondrial function and inhibiting apoptosis via PHLDA2 downregulation, while mitigating inflammatory pathways via Tnfaip8l3 suppression. Conversely, the alteration in Syt13 underscores the importance of monitoring tubular function (e.g., NaCl reabsorption) during neonatal NMN administration.

Notably, these specific molecular adaptations were not accompanied by histological evidence of renal injury, aligning with prior reports that NMN can ameliorate mitochondrial dysfunction and cellular damage in acute kidney injury (Liang J. et al., 2024). We hypothesize that this dissociation between transcriptomic changes and tissue phenotype highlights the dose- and regimen-dependent nature of NMN’s effects. Chronic, low-dose NMN may exert clear protective benefits by sustained enhancement of NAD^+^-dependent pathways. In contrast, the acute, high-dose exposure in this study likely triggered a transient stress response and compensatory adaptation at the transcriptional level—sufficient to maintain homeostasis without crossing the threshold to overt structural damage. This observation further underscores the high acute safety margin of NMN at the tested dose.

### 4.14 Impact of NMN on brain neuron morphology

To evaluate the potential effects of high-dose NMN on the neonatal brain, a systematic morphological analysis was performed on brain tissues from control and NMN-treated neonatal mice. Histopathological examination (H&E staining) revealed that neurons in both groups were densely packed and orderly arranged, with uniform cellular morphology and evenly distributed chromatin. No signs of edema, necrosis, or inflammation were observed. Immunofluorescence staining for the neuronal marker NeuN confirmed no significant differences in neuronal morphology or distribution between groups. In NMN-treated brains, tau protein was appropriately enriched in axonal regions, and MAP2 displayed a clear, fasciculated distribution around neuronal somata, exhibiting typical cytoskeletal architecture without evidence of axonal breakage or dendritic damage.

### 4.15 Reconciling the findings with prior axon injury models

This finding appears to contrast with a report by Di Stefano et al., which identified NMN accumulation as an early, key event triggering axonal degeneration in isolated neurons and sciatic nerve injury models (Di Stefano et al., 2015). We posit that this discrepancy arises from fundamental differences in the experimental models and underlying pathophysiological states. Di Stefano’s work focuses on the specific pathology of “Wallerian-like degeneration” following acute axonal injury (e.g., axotomy), whereas our study examines structurally intact, normal neonatal neurons. A pivotal mechanistic distinction is the status of nicotinamide mononucleotide adenylyltransferase (NMNAT). In injury models, NMNAT functional impairment or insufficient activity leads to pathological NMN accumulation, which cannot be efficiently converted to NAD^+^, thereby activating pro-degenerative pathways. Conversely, in healthy neurons with fully functional NMNAT—as in our model—exogenous NMN is readily metabolized to replenish the NAD^+^ pool, supporting neuroprotective and metabolic functions. This highlights NMN’s context-dependent “double-edged sword” effect in the brain: a beneficial NAD^+^ precursor when NMNAT is intact, but a direct promoter of axonal degeneration in settings of NMNAT deficiency.

In summary, this study did not observe NMN-induced neuromorphological damage in the normal neonatal brain, a finding that does not conflict with conclusions from injury models but rather underscores the situational specificity of NMN’s biological effects. This insight carries direct clinical relevance: future applications of NMN, particularly for conditions involving potential neural injury, should consider evaluating patient NMNAT levels or activity to ensure the compound exerts its beneficial role within a permissive metabolic environment and to mitigate potential risks.

### 4.16 Brain transcriptomic profiling and mechanistic exploration

To evaluate the potential molecular impact of high-dose NMN on the neonatal central nervous system, high-throughput RNA sequencing was performed on brain tissues from control and NMN-treated neonatal mice. The analysis identified seven differentially expressed genes (DEGs) in the treatment group (screening criteria: |log_2_FC| > 1 and P < 0.05). GO and KEGG enrichment analyses revealed that these DEGs were significantly clustered in biological processes and pathways associated with nervous system homeostasis and cellular development.

Among these, three key DEGs—glial fibrillary acidic protein (GFAP), toll-like receptor 2 (TLR2), and growth hormone (GH)—were of particular interest, as their altered expression may reflect NMN’s potential to modulate pathways involved in neuroinflammation and neurodevelopment.

### 4.17 Mechanistic implications of altered GFAP and TLR2 expression

The downregulation of GFAP (glial fibrillary acidic protein) and TLR2 (toll-like receptor 2) in the neonatal brain following NMN exposure points to a coordinated attenuation of neuroinflammatory pathways.

### 4.17.1 GFAP downregulation and neuroinflammation

The observed downregulation of the GFAP gene, which encodes the principal intermediate filament protein of astrocytes, suggests that NMN may attenuate astrocyte reactivity and associated neuroinflammation. Although direct mechanistic evidence is limited, emerging clinical research indicates that plasma GFAP levels correlate with synaptic loss and serve as a prognostic marker (Wang et al., 2025). Therefore, monitoring plasma GFAP could provide a translational reference for assessing NMN’s neural safety and efficacy. Collectively, GFAP downregulation points to NMN’s potential to reduce neuroinflammation and help maintain synaptic homeostasis.

### 4.17.2 TLR2 downregulation and innate immune signaling

TLR2 is a key pattern-recognition receptor in the innate immune system. Its activation on microglia triggers neuroinflammatory cascades that can lead to synaptic dysfunction and loss (Freria et al., 2012; La Vitola et al., 2018). Conversely, TLR2 knockout enhances neuronal resilience to toxic stimuli and promotes synaptic plasticity (Gorup et al., 2019; Suh et al., 2013). The suppression of TLR2 expression by NMN strongly indicates a mechanism through which NMN may mitigate neuroinflammation and protect synaptic integrity by inhibiting the TLR2-mediated pathway.

### 4.17.3 Mechanistic implications of coordinated GFAP and TLR2 downregulation

The simultaneous downregulation of GFAP (glial fibrillary acidic protein) and TLR2 (toll-like receptor 2) suggests a concerted anti-neuroinflammatory mechanism through which NMN may confer neuroprotective benefits. This dual suppression likely dampens both astrocyte and microglia-driven inflammatory responses, fostering a microenvironment conducive to synaptic stability and function.

The decrease in GFAP, a canonical marker of astrocyte activation, directly indicates a reduction in astrocytic reactivity (Linnerbauer et al., 2020). TLR2, a key upstream activator of the NF-κB signaling pathway, is primarily expressed in microglia; its downregulation reflects an inhibition of NF-κB pathway activity (Kuang et al., 2024). Mechanistically, SIRT1 is known to inhibit NF-κB transcriptional activity by deacetylating its p65 subunit. Therefore, NMN, by elevating NAD^+^ levels and activating SIRT1, may suppress NF-κB signaling, leading to the downstream reduction of both TLR2 and GFAP expression.

These transcriptomic changes collectively confirm NMN’s inhibitory effect on core neuroinflammatory pathways. Furthermore, the synergistic downregulation of TLR2 (microglial) and GFAP (astrocytic) suggests that NMN may also modulate astrocyte reactivity indirectly by first regulating the immune state of microglia, highlighting an integrated glial response.

### 4.18 Mechanistic implications of upregulated GH expression

Growth hormone (GH) is not only a pivotal regulator of somatic growth but also a significant neuroactive factor, playing indispensable roles in central nervous system development, repair, and cognitive function (Devesa et al., 2013; Scheepens et al., 2005). This study found that NMN upregulates GH expression in the neonatal brain. Mechanistically, this may involve the SIRT1-GH axis: SIRT1, an NAD^+^-dependent deacetylase, participates in the transcriptional regulation of GH synthesis in the hypothalamus and pituitary, and maintains functional connectivity with growth hormone receptor (GHR)-expressing neurons (Fedorczak et al., 2023). Therefore, NMN may elevate NAD^+^ levels, enhance SIRT1 activity, and subsequently promote cerebral GH expression, thereby exerting neurodevelopmental and protective effects.

### 4.19 Integrated interpretation and developmental specificity

At the transcriptomic level, we observed that NMN concomitantly modulates genes involved in neuroinflammation (downregulation of GFAP and TLR2) and neurodevelopment (upregulation of GH). This pattern aligns with prior findings in adults, where NMN has demonstrated potential in improving metabolism, antioxidant defense, and neuroprotection (Liang N. et al., 2024; Ma et al., 2024).

However, the specific functional significance of the gene expression profile elicited in neonatal mice—including other tissue-specific changes such as those in Fbf1 and Ptk6 in the liver—may differ from that in adults. This underscores the developmental stage-dependent nature of the biological effects of NMN, highlighting that responses to NAD^+^ precursor intervention are shaped by the unique metabolic and regulatory landscape of the developing organism.

### 4.20 Neonatal-specific transcriptomic landscape and adaptive mechanisms underlying high-dose NMN exposure

To delineate the core mechanisms and biological effects of high-dose NMN in neonates, we conducted a high-throughput transcriptome analysis. While adult studies largely affirm the safety of conventional NMN doses (Song et al., 2023), this work focused on acute limit testing in the unique neonatal physiological context. Critically, even at a high dose (5,000 mg/kg), no significant histopathological damage was observed. However, specific and pathway-enriched transcriptomic alterations were evident, suggesting that newborns—with their immature metabolic and immune systems— exhibit a unique transcriptional responsiveness to NMN, eliciting a fine molecular adaptive response distinct from adults. These changes likely represent compensatory adjustments to maintain homeostasis under metabolic load rather than indicators of direct toxicity, providing essential molecular insights for a comprehensive clinical safety evaluation.

### 4.21 A unified core mechanism: systemic SIRT1 activation

Transcriptomic profiling indicates that NMN systemically activates the NAD^+^-dependent deacetylase SIRT1 and its downstream pathway. The NAD^+^ surplus provided by high-dose NMN enhances SIRT1 activity, establishing it as the common upstream regulator orchestrating the observed organ-specific molecular changes and extensively modulating signaling networks related to cellular homeostasis.

### 4.22 Highly coordinated multi-organ transcriptomic responses

Building upon this core mechanism, we observed highly synergistic transcriptomic changes across the liver, kidney, and brain, which converge on three principal adaptive themes.

We first observed a systemic anti-inflammatory trend was evident, marked by the downregulation of inflammation- or immune-related genes: tyrosine kinases (Srms, Ptk6) in the liver, Tnfaip8l3 in the kidney, and TLR2 along with the astrocytic marker GFAP in the brain. These concerted changes consistently point to the systematic suppression of the NAD^+^/SIRT1/NF-κB signaling axis. By deacetylating the NF-κB p65 subunit, SIRT1 inhibits its transcriptional activity, thereby reducing the basal inflammatory tone across multiple organs—a key adaptive response to maintain homeostasis under high metabolic load.

Furthermore, extensive adjustments to the cytoskeleton and membrane transport systems were revealed. This includes alterations in cytoskeleton-related genes (Krt222, Fbf1) in the liver, downregulation of the vesicle transport gene Syt13 in the kidney, and reduction of GFAP in the brain. Collectively, these changes highlight the widespread impact of NMN on cellular mechanical structures and membrane trafficking networks, likely representing structural and functional compensation initiated by cells to adapt to rapid metabolic state shifts following NAD^+^/SIRT1 axis activation.

Simultaneously, the data suggest a potential modulation of gene programs during a critical developmental window. In the developing neonatal model, concurrent expression changes were detected in the liver epithelial differentiation-related gene Ptk6, the kidney organ development-related imprinted gene PHLDA2, and the brain neurodevelopmental factor GH. This pattern indicates that high-dose NMN exposure may influence specific gene expression programs active during this vulnerable period, aligning with prior reports on perinatal NMN exposure and neurobehavioral development (Ear et al., 2019).

A crucial finding is that none of these transcriptomic changes were enriched for classic injury or cell death pathways (e.g., apoptosis, necrosis). Instead, they uniformly pointed toward biological processes aimed at maintaining homeostasis. This perfectly explains, at a molecular level, the absence of tissue damage upon histopathological examination: under acute high-dose exposure, NMN primarily triggers a series of subtle, compensatory physiological adjustments rather than direct cytotoxic damage.

### 4.23 Significance and context of the neonatal acute limit test

The value of this neonatal-specific acute limit toxicity test (5,000 mg/kg, ∼100x the conventional dose) is paramount. While it confirms a wide acute safety window based on the lack of histomorphological injury, this conclusion requires careful interpretation within the context of unique neonatal pharmacokinetics. The limiting dose reveals the essence of “metabolic stress” rather than “direct toxicity.” When corrected for body surface area (CDER, 2005) and the low metabolic clearance rate of newborns (30%–60% of adults), the administered dose translates to an “effective exposure load” equivalent to 47–94 g of NMN for an adult, constituting a prototypical extreme metabolic stress test model.

In this model, sustained exposure to supra-physiological levels of NAD^+^ and its metabolites triggers a transcriptional reprogramming aimed at multi-organ coordination and homeostasis maintenance. All observed adaptive changes are fundamentally rooted in the immature drug metabolism and clearance systems of the newborn. Therefore, the safety profile of NMN cannot be simply extrapolated from adult data. The unique gene expression profile elucidated here serves as direct evidence of this developmental-stage specificity, underscoring the critical necessity of conducting neonatal-specific toxicological assessments for a complete and accurate safety evaluation.

### 4.24 A dual-perspective safety assessment of NMN in neonates and its clinical translation implications

This study defines the safety profile of NMN during the neonatal period from two complementary perspectives. At the traditional histopathological level, NMN demonstrates a very low intrinsic risk of direct toxicity. Concurrently, at the level of fine molecular regulation, high-dose exposure triggers significant yet adaptive transcriptional reprogramming, primarily involving metabolic and signaling adjustments aimed at maintaining homeostasis.

These findings provide a dual foundation for its potential future application in neonatal medicine. They not only confirm the compound’s low acute toxicological risk but also emphasize the necessity to explore a safe and effective therapeutic window guided by developmental pharmacology principles, under appropriate monitoring.

Based on these insights, this study offers prudent preliminary recommendations for the potential clinical use of NMN in neonates. Furthermore, the observed multi-organ transcriptomic signatures, indicative of coordinated anti-inflammatory and adaptive responses, reveal its potential for multi-organ protective effects. Collectively, this work provides a crucial and nuanced evidence base for advancing the safe translation of NMN into neonatal care.

### 4.25 Clinical recommendations based on multi-organ transcriptomic insights

Based on the specific adaptive and regulatory responses triggered by high-dose NMN exposure at the transcriptomic level in the liver, kidney, and brain, the following clinical recommendations are proposed to ensure safety in special physiological conditions.

Regarding the liver, caution is warranted when considering administration to individuals with pre-existing hepatocellular injury. It is advisable to monitor parameters related to liver hemodynamics and biliary excretion function, which may serve as indirect indicators of potential cytoskeletal and mechanical alterations within the organ. For the kidneys, close monitoring of tubular function is essential, with a particular focus on metrics such as the NaCl reabsorption rate to evaluate the renal tubules’ adaptive capacity to metabolic load. In the context of the nervous system, peripheral (e.g., plasma or CSF) levels of glial fibrillary acidic protein (GFAP) could be considered a useful reference biomarker for indirectly assessing neuroinflammatory status and glial cell activation.

### 4.26 Integrated summary: Safety profile and therapeutic potential of NMN in neonates

This study elucidates the dual facets of high-dose NMN exposure in neonates. Beyond the primary safety assessment, the molecular analysis reveals potential beneficial effects of NMN, suggesting that its role extends beyond risk mitigation to active biological modulation.

The observed transcriptomic changes point to multi-organ protective potential. This includes potential anti-tumor and cytoprotective effects, such as the inhibition of liver Ptk6 expression hinting at a potential anti-hepatoma activity, and the downregulation of renal PHLDA2, which is associated with improved mitochondrial function and suppressed apoptosis. Concurrently, effects indicative of promoted neural development and repair were evident: the upregulation of cerebral GH expression suggests a pro-neurodevelopmental capacity, while the downregulation of TLR2 and GFAP underscores potent anti-inflammatory and synaptic protective properties.

In summary, through a limit-test model, this work systematically evaluates the acute safety of NMN in the neonatal period, integrating assessments from histomorphology to deep molecular mechanisms. Based on the specific molecular clues uncovered, it proposes targeted clinical monitoring strategies. These findings provide a crucial experimental foundation for supporting NMN as a potential therapeutic agent for neonatal diseases, while also offering prospective theoretical guidance for refining its clinical application and safety management in the future.

### 4.27 Study limitations and future directions

This study employed a high-dose acute exposure model to systematically evaluate the potential toxicity of NMN in neonatal mice, generating significant insights across histopathology, serum biochemistry, and multi-organ transcriptomics. While the experimental design was rigorous, we acknowledge several inherent limitations, which also delineate clear pathways for future investigation.

### 4.27.1 Limitations in sample size and administration protocol

As a preliminary acute limit test, the study focused on verifying the safety threshold at a single high dose, without incorporating multi-dose gradients or exploring alternative administration routes (e.g., oral). Consequently, while the results robustly confirm the low direct toxicity of NMN under extreme conditions, they do not establish a complete dose-response relationship or identify an optimal administration method. These critical parameters necessitate further investigation in subsequent studies with expanded sample sizes.

### 4.27.2 Lack of extended observation and dynamic monitoring

To ensure endpoint consistency and avoid interference from the inherent metabolic immaturity of neonates, serum biochemical analyses were concentrated at a single time point (day 14). Although this aligns with histological and transcriptomic endpoints, this design may not capture earlier, transient fluctuations in key biomarkers. Future studies will incorporate dynamic monitoring at multiple early time points to construct a more comprehensive pharmacokinetic and pharmacodynamic profile of the neonatal response.

### 4.27.3 Scope of the research focus

The present study primarily concentrated on acute tissue-level morphology and initial molecular responses. It did not evaluate subacute or chronic toxicity, long-term impacts on growth and development (developmental toxicity), or complex functional outcomes such as neurobehavioral effects. These aspects are paramount for clinical translation and will be the subject of systematic, independent long-term experiments.

### 4.27.4 Stages of clinical translation

This study provides crucial preclinical proof-of-concept and preliminary safety data in a neonatal mouse model. However, extrapolating these findings to human neonates requires extreme caution and must be preceded by validation through an extended chain of evidence, including studies in other species and dedicated juvenile animal models.

It is important to emphasize that the aforementioned limitations are characteristic of exploratory preclinical research and do not diminish the core contributions of this work. By innovatively utilizing an extreme stress-test model, this study revealed the high histological safety of NMN and the adaptive transcriptional reprogramming across multiple organs in neonates. These findings not only empirically demonstrate NMN’s low direct toxicity but also provide indispensable foundational evidence and a clear research roadmap for future efforts aimed at defining safe neonatal dosages, elucidating deeper mechanisms, and formulating rational clinical monitoring strategies.

## Data Availability

The data presented in the study are deposited in the SRA repository, accession number PRJNA1461357 (https://www.ncbi.nlm.nih.gov/bioproject/PRJNA1461357).
